# Highly Efficient Single-Step Enrichment of Low Abundance Phosphopeptides from Plant Membrane Preparations

**DOI:** 10.3389/fpls.2017.01673

**Published:** 2017-09-27

**Authors:** Xu Na Wu, Lin Xi, Heidi Pertl-Obermeyer, Zhi Li, Liang-Cui Chu, Waltraud X. Schulze

**Affiliations:** Department of Plant Systems Biology, University of Hohenheim, Stuttgart, Germany

**Keywords:** membrane protein, phosphorylation, proteomics, kinase substrate relationship, nitrate signaling

## Abstract

Mass spectrometry (MS)-based large scale phosphoproteomics has facilitated the investigation of plant phosphorylation dynamics on a system-wide scale. However, generating large scale data sets for membrane phosphoproteins usually requires fractionation of samples and extended hands-on laboratory time. To overcome these limitations, we developed “ShortPhos,” an efficient and simple phosphoproteomics protocol optimized for research on plant membrane proteins. The optimized workflow allows fast and efficient identification and quantification of phosphopeptides, even from small amounts of starting plant materials. “ShortPhos” can produce label-free datasets with a high quantitative reproducibility. In addition, the “ShortPhos” protocol recovered more phosphorylation sites from membrane proteins, especially plasma membrane and vacuolar proteins, when compared to our previous workflow and other membrane-based data in the PhosPhAt 4.0 database. We applied “ShortPhos” to study kinase-substrate relationships within a nitrate-induction experiment on *Arabidopsis* roots. The “ShortPhos” identified significantly more known kinase-substrate relationships compared to previous phosphoproteomics workflows, producing new insights into nitrate-induced signaling pathways.

## Introduction

Protein phosphorylation is regulated by protein kinases and phosphatases. These enzymes are key players in cellular signal transduction pathways catalyzing phosphorylation or dephosphorylation of target proteins and thereby modulating kinase signaling cascades (Hrabak et al., 2003; Pitzschke et al., 2009; Weinl and Kudla, 2009; Asano et al., 2012; Meng and Zhang, 2013), membrane transport (Liu and Tsay, 2003; Tornroth-Horsefield et al., 2006; Lanquar et al., 2009; Rudashevskaya et al., 2012; Fuglsang et al., 2014), and metabolic activities (Sanchez and Heldt, 1990; Mcmichael et al., 1993; Huber and Huber, 1996). Thus, identification and quantitation of proteins phosphorylated under specific cellular conditions is a critical for the understanding of the regulatory principles of these signal transduction, transport and metabolic processes.

Today, mass spectrometry (MS)-based phosphoproteomics has become a routine procedure to globally study the dynamics of phosphorylation in plants in context of nutrient stimulation (Nühse et al., 2003; Niittylä et al., 2007; Tran and Plaxton, 2008; Engelsberger and Schulze, 2012; Lan et al., 2012), defense (Benschop et al., 2007), abiotic stress responses (Xue et al., 2013; Stecker et al., 2014), primary metabolism (Reiland et al., 2009; Reiland et al., 2011) or hormone signaling (Chen et al., 2010; Zhang et al., 2013; Lin et al., 2015). Plant membrane proteins, especially those located at the plasma membrane (PM), are involved in the perception of external signals and in regulation of the initial response through receptor kinases (Chinchilla et al., 2007; Kim et al., 2009; Marshall et al., 2012; Osakabe et al., 2013).

The phosphorylation status of kinases and their substrates is of high interest when studying signaling pathways. Many protein kinases are regulated by autophosphorylation or by upstream kinases in signaling cascades (Zulawski et al., 2013; Zulawski and Schulze, 2015). Kinase substrates such as transporters, transcription factors or metabolic enzymes are modulated in response to external or internal stimuli and result in adaptive phenotypes (Czechowski et al., 2005; Chinchilla et al., 2007). MS-based phosphoproteomics is a widespread technology used to investigate protein kinase substrates on a large scale (Reiland et al., 2009; Wang et al., 2013; Li et al., 2014; Lin et al., 2015; Roitinger et al., 2015). Thereby it is of particular interest to identify regulatory kinase-substrate pairs. However, the knowledge of kinase-substrate relationships is not complete, as for many kinases the substrates are still unknown, and for many substrates, the respective kinases are not known.

The analysis of phosphorylated peptides by MS has undergone a continuous optimization, but specific technical challenges remain, such as selective suppression of phosphopeptide ions in presence of other peptides, lower ionization efficiencies and increased hydrophobicity with associated tendencies for higher losses during reversed-phase chromatography (Steen et al., 2006). Therefore, in the past years, various protocols for phosphopeptide enrichments were developed using chelating and complexing agents (Larsen et al., 2005; Nühse and Peck, 2006; Sugiyama et al., 2007; Thingholm et al., 2008; Nakagami, 2014). Thereby, the addition of quenching agents, such has dihydroxybenzoic acid, lactic acid or glycolic acid proved important to reduce the enrichment of non-phosphopeptides with acidic amino acids (Larsen et al., 2005; Sugiyama et al., 2007). Most of these workflows were initially developed for whole cell extracts in combination with multiple fractionation of the extracts to increase phosphopeptide coverage (Mohammed and Heck, 2010; Li et al., 2012).

However, despite the great variety of phosphopeptide enrichment protocols three major technical obstacles still pose a challenge for efficient phosphoproteome research of membrane proteins (Li et al., 2008). Firstly, the analysis of plant membrane signaling proteins is inherently more difficult if total cell extracts are used, since membrane proteins are usually of low abundance in these extracts and most of the membrane proteins are discarded with the cell wall debris. Secondly, in a complex peptide mixture after protein digestion phosphopeptides are present in lower abundance compared to unmodified peptides. Thirdly, despite advances in instrument technology, it remains difficult to specifically identify phosphopeptides of membrane proteins within the overabundance of non-phosphopeptides in a complex peptide mixture due to the physicochemical properties of phosphopeptides (Steen et al., 2006; Nakagami et al., 2010). Therefore, high quality plant membrane protein extraction in combination with highly efficient phosphopeptide enrichment methods prior to mass spectrometric analysis are critical steps for maximal identification of membrane associated phosphopeptides.

**FIGURE 1:**
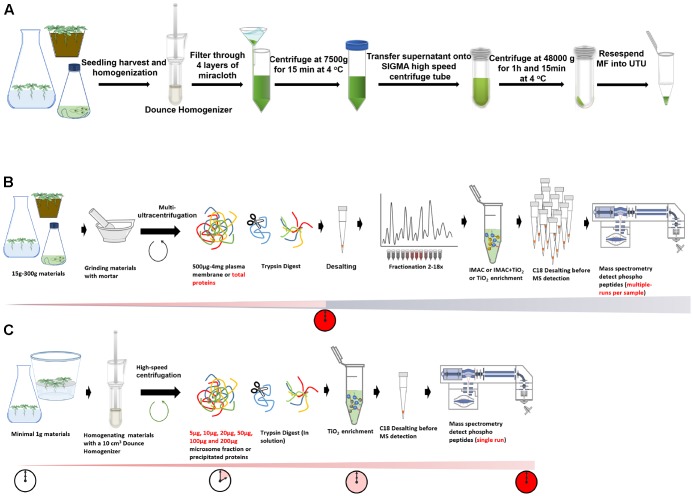
Fast and cost-effective ShortPhos phosphoproteomic workflow with single run analysis of phosphoproteome compared with conventional workflow. **(A)** An overview of the general procedure of optimized MF isolation method. **(B)** Conventional phosphoproteomics workflow usually requires 500–4000 μg of plasma membrane (PM) for protein digestion, peptides desalting, peptides fractionation, phosphopeptides enrichment by TiO2 or IMAC and multiple LC-MS/MS runs per sample, resulting in slow and costly workflow in large scale experiment. **(C)** “ShortPhos” phosphoproteomic workflow described in this paper requires minimal microsomal fraction (MF) for protein digestion, no desalting or fractionation before phosphopeptide enrichment, and a single run of LC-MS/MS analysis per sample.

In a conventional workflow, the analysis of the plant PM phosphoproteome requires 15–300 g of frozen seedlings or plant cells which usually are ground into a fine powder by using a mortar and pestle. Then, 500–4000 μg of PM, purified from microsomal pellets using a two-phase system of dextran and polyethylene glycol and multiple ultracentrifugation steps (Nühse et al., 2003; Kierszniowska et al., 2009; Engelsberger and Schulze, 2012; Szymanski et al., 2013), is processed by trypsin digestion. This is then usually followed by peptide desalting and a fractionation of peptides using strong cation (Mohammed and Heck, 2010) or anion exchanger (Li et al., 2012). Phosphopeptides are then enriched by immobilized metal ion chromatography (IMAC) (Nühse et al., 2003, 2004; Niittylä et al., 2007; Whiteman et al., 2008a,b; Szymanski et al., 2013), titanium dioxide (TiO_2_) (Benschop et al., 2007; Niittylä et al., 2007), a combination of IMAC and TiO_2_ (Reiland et al., 2009; Mayank et al., 2012) or other complexing ions (Zhou et al., 2013). Finally, multiple LC-MS/MS runs per sample are performed (Nühse et al., 2003; Benschop et al., 2007; Engelsberger and Schulze, 2012; Mayank et al., 2012). Notably, such workflows take a very long hand-on sample preparation time, result in extended instrument operation time, and are thus rather expensive, especially for a large number of samples (**Figure 1B**). Moreover, it is difficult to prepare enough PM for these conventional phosphoproteome studies when starting material is limited. Therefore, using the microsomal fraction (MF) for analyses became much more attractive to overcome these limitations (Wu and Schulze, 2015). In addition, a phosphoproteomic analysis of MF also helps to understand phosphorylation signaling in endomembranes, such as the ER, Golgi apparatus and the vacuole. In existing protocols applying IMAC or TiO_2_ (Titanium dioxide)-based phosphopeptide enrichment without strong cation exchange fractionation to MF preparations (Wu et al., 2013, 2014), rather low yields of phosphopeptides were achieved, especially from proteins with low abundances.

We therefore developed a new workflow, named “ShortPhos” which highly improves the phosphoproteome coverage (up to 85%) from plant membrane proteins in a simple, fast, and highly reproducible procedure without requiring any additional fractionation (**Figure 1C**).

## Materials and Methods

### Plant Material

For experiments optimizing the phosphopeptide enrichment, *Arabidopsis* seeds of wild type (col-0) were germinated and grown under 16/8 h day/night (22 °C, 120 μE/s^∗^m^2^) in aaa MS medium plus 0.5% w/v sucrose in flasks for 2 weeks. Whole seedlings were harvested for microsomal protein preparation. For nitrate starvation-resupply experiments, seedlings were germinated and grown into JPL-medium composed of micro- and macronutrients (Jouanneau and Peaud-Lenoel, 1967), with a total of 1 mM nitrogen and supplemented with 0.5% w/v sucrose. After 2 weeks, seedlings were starved of nitrogen for 2 days by changing the growth medium to a nitrogen-free medium. Nitrate was then resupplied to a final concentration of 0.2 and 5 mM for 15 min before harvesting roots for microsomal protein preparation. All experiments consisted of at least three biological replicates.

### Preparation of Microsomal Fraction

A total of 1 g of frozen seedlings or roots (fresh weight) was broken into small pieces and homogenized in 10 ml extraction buffer (330 mM sucrose, 100 mM KCl, 1 mM EDTA (Ethylenediaminetetraacetic acid disodium salt dihydrate), 50 mM Tris-MES, fresh 5 mM DTT (Dithiothreitol), and 1 mM PMSF (phenylmethylsulfonyl fluoride), pH 7.5) (Pertl et al., 2001) in the presence of 0.5% v/v proteinase inhibitor mixture (Sigma–Aldrich, Germany) and phosphatase inhibitors (25 mM NaF, 1 mM Na_3_VO_4_, 1 mM benzamidin, 3 μM leupeptin) in a Dounce Homogenizer. At least 50 strokes were performed. The homogenate was filtered through four layers of miracloth and centrifuged for 15 min at 7500 × g at 4°C. The pellet was discarded, and the supernatant was centrifuged for 75 min at 48,000 × *g* at 4°C. The microsomal pellet was re-suspended in 100 μl UTU (6 M urea, 2 M thiourea, pH 8). Protein concentrations were determined using a Bradford (Sigma–Aldrich, Germany) assay with BSA (Bovine serum albumin) as protein standard. Samples were stored at -80°C.

### In Solution Trypsin Digestion

In dilution experiment, 5–200 μg MF from seedlings were aliquoted into tubes. In nitrate starvation-resupply experiment, 100 μg MF from roots were aliquoted into tube. Corresponding volumes of DTT reduction buffer (1.3 μl of 5 mM DTT reduction buffer stock solution for every 50 μg protein) were added into samples and incubated at room temperature for 30 min, followed by an incubation with alkylation buffer (1 μl of 27mM iodoacetamide alkylation buffer stock solution for every 50 μg protein) for 20 min. MF samples were predigested for 3 h with endoproteinase Lys-C (1 μl of 0.5 μg/μl stock solution for every 50 μg protein; Wako Chemicals, Neuss, Germany) at room temperature. After four-fold dilution with 10 mM Tris-HCl (pH 8), samples were digested with sequencing-grade modified trypsin (1 μl of 0.5 μg/μl stock solution for every 50 μg protein; Promega, Germany) overnight at 37°C. After overnight digestion, 10% v/v trifluoroacetic acid (TFA) was added (until the pH was 3 or less) to stop the digestion. Digested peptides were dried in a vacuum concentrator and dissolved in 200 μl of 1 M glycolic acid in 80% v/v acetonitrile (ACN) and 5% v/v trifluoroacetic acid (TFA) before phosphopeptide enrichment over titanium dioxide (TiO_2_) (GL Sciences, Japan).

### TiO2 Phosphopeptide Enrichment

Phosphopeptides were enriched over TiO_2_ (Titanium dioxide). A ratio of 10:1 TiO_2_ beads/protein were resuspended into 100 μl methanol and centrifuged at 2,500 × *g* at room temperature for 2 min to discard methanol. TiO_2_ beads were subsequently washed once with 100 μl of 1% v/v ammonia solution for 10 min with vortex mixing and centrifuged at 2,500 × *g* at room temperature for 2 min to discard solution. TiO_2_ beads were then equilibrated with 50 μl of 1 M glycolic acid in 80% v/v ACN and 6% v/v TFA for 60 s and centrifuged at 2,500 × *g* at room temperature for 2 min to discard solution. Two hundred microliter digested peptides were mixed with equilibrated TiO_2_ beads for 30 min incubation with continuous mixing and centrifuged at 2,500 × *g* at room temperature for 2 min to discard solution. Peptides and TiO_2_ beads mixture were washed once with 100 μl of 1 M glycolic acid in 80% v/v ACN and 6% v/v TFA for 30 s and centrifuged at 2,500 × *g* at room temperature for 2 min to discard solution. Peptides and TiO_2_ beads mixture were then washed two times with 100 μl of 80% v/v ACN and 1% v/v TFA for 2 min and centrifuged at 2,500 × *g* at room temperature for 2 min to discard solution. Enriched peptides were eluted from TiO_2_ beads three times with 1% v/v ammonia solution and 15 min incubation. Eluates were immediately acidified with 70 μl of 10% v/v formic acid.

### Peptides Desalting with C18 Stage Tips

Prior to mass spectrometric analysis enriched peptides were desalted over a C18 stage tip as described previously (Rappsilber et al., 2003). C18 stage tips were equilibrated with 50 μl of 80% v/v ACN and 0.1% v/v TFA and washed two times with 100 μl of 5% v/v ACN and 0.1% v/v TFA. Eluates (enriched phosphopeptides) were loaded onto equilibrated C18 stage tip and washed two times with 100 μl of 5% v/v ACN and 0.1% v/v TFA and then eluted with two times 20 μl of 80% v/v ACN and 0.1% v/v TFA.

### LC-MS/MS Analysis

Enriched peptides were resuspended in 5 μl resuspension buffer (0.2% v/v TFA, 5% v/v ACN) and analyzed via LC-MS/MS using standard setting as described in **Supplementary Table S1** with nanoflow Easy-nLC (Thermo Scientific, Germany) as an HPLC system and an Orbitrap hybrid mass spectrometer (Q-Exactive, Thermo Scientific, Germany) as a mass analyzer. Peptides were eluted from a 75 μm analytical column (EasySpray, Thermo Scientific, Germany) on a linear gradient running from 5 to 90% acetonitrile over 135 min and sprayed directly into the Q-Exactive mass spectrometer. Peptides were identified via MS/MS based on the information-dependent acquisition of fragmentation spectra of multiple charged peptides. Up to 12 data-dependent MS/MS spectra were acquired for each full-scan spectrum acquired at 70,000 full-width at m/z 400 resolution.

### Protein Identification and Ion Intensity Quantitation

Raw data acquired by the mass spectrometer were processed using MaxQuant, version 1.5.3.8 (Cox and Mann, 2008) using setting as described in **Supplementary Table S2** for protein identification and ion intensity quantitation. Spectra were matched against the Arabidopsis proteome (TAIR10, 35,386 entries) using Andromeda (Cox et al., 2011). Common contaminants (trypsin, keratin, etc.) were included during database searches. Carbamidomethylation of cysteine was set as a fixed modification, and the oxidized methionine (M), acetylation (protein N-term) and phosphorylation (STY) were set as variable modifications. Trypsin was specified as the digseting protease, and up to two missed cleavages were allowed. The mass tolerance for the database search was set to 20 ppm for full scans and 0.5 Da for fragment ions. The multiplicity was set to 1. For label-free quantitation, retention time matching between runs was chosen within a time window of 1 min. False discovery rate cutoffs were set to 0.01 for peptide and protein identification, and to 0.05 for phosphorylation site assignment. The location of phosphorylation sites was determined by the site-scanning algorithm within Andromeda. Hits to contaminants (e.g., keratins) and reverse hits identified by MaxQuant were excluded from further analysis. The identified peptides including non-phosphopeptdes and phosphopeptides (**Supplementary Table S8**) were processed further. Phosphopeptides, including their spectra, were submitted to the phosphorylation site database PhosPhAt 4.0 and are publicly available.

### Data Analysis

Bioinformatics analysis was performed with Perseus, Microsoft Excel, SigmaPlot. Annotations were extracted from MapMan (Thimm et al., 2004), subcellular locations were obtained from SUBA3 (Tanz et al., 2013).

## Results and Discussion

The goal of this study was to set up an efficient, fast, simple and cost-effective workflow for phosphoproteome studies on plant membrane proteins, especially using a small amount of starting material. The workflow is applicable to plant cell cultures, liquid culture seedlings, soil grown plants, or sectioned tissue materials. To address this, we adjusted two critical steps: (1) the MF preparation method was optimized for small amounts of starting material, and (2) a more efficient and simple phosphopeptide enrichment method was developed, which improved the coverage and reproducibility of phosphopeptide quantification in single MS runs. Overall the new workflow has potential of saving both time and expenses.

### Workflow Optimization

We firstly optimized membrane protein extraction using liquid-grown *Arabidopsis* wild type seedlings by choosing Dounce Homogenizer-based MF isolation (Pertl-Obermeyer and Obermeyer, 2014) instead of conventional extraction of MF and PM from mortar and pestle ground material (**Figures 1A,C**). Tissue disruption by use of a Dounce homogenizer generally is considered gentler to membrane integrity. Thus membrane vesicle formation in the extraction buffer is more efficient compared to extracts from tissue disrupted by mortar and pestle. This resulted in higher yields of membrane proteins from less starting material (e.g., 1 g of tissue). On average, around 500 μg of MF protein was obtained from 1 g starting plant material (**Supplementary Table S3**). Conventional methods for MF isolation from frozen powder required 4 g frozen powder and only yielded around 300 μg MF protein (Wu et al., 2013, 2014). The processing time for 6 parallel samples took around 2 h (**Figure 1A**). Isolated MF was directly resuspened in 6 M urea and 2 M thiourea pH 8 (UTU), which is a commonly used solution for protein denaturation before in-solution trypsin digestion (Olsen et al., 2004).

Secondly, the processing of phosphopeptide enrichment was optimized (**Figure 1C**): (1) Peptide desalting was eliminated after trypsin digestion. This step is traditionally performed before phosphopeptide enrichment (Nakagami, 2014), but resulted in a loss of tryptic peptides including phosphopeptides. (2) We do not use any peptide fractionation. Thus, we performed a single MS run per sample (on extended LC-gradients), which saved instrument time and expenses. (3) We chose Titanium dioxide (TiO_2_) for phosphopeptide enrichment because it displayed the greatest advantages in handling and gave highest enrichment specificity for phosphopeptides (Pinske et al., 2004; Sugiyama et al., 2007; Thingholm et al., 2008). In total, sample preparation took less than 2 days for 10–12 samples using the “ShortPhos” workflow and mass spectrometric analysis over a 2-h MS gradient. The sample preparation and mass spectrometric analysis of the same amount of samples using our previous workflow with 10 fractions per sample would have taken at least 8 days (**Figures 1B,C**). Thus, the “ShortPhos” workflow resulted in less hands-on time compared to the conventional workflows used in previous research (Niittylä et al., 2007; Engelsberger and Schulze, 2012; Wu et al., 2013, 2014).

**FIGURE 2:**
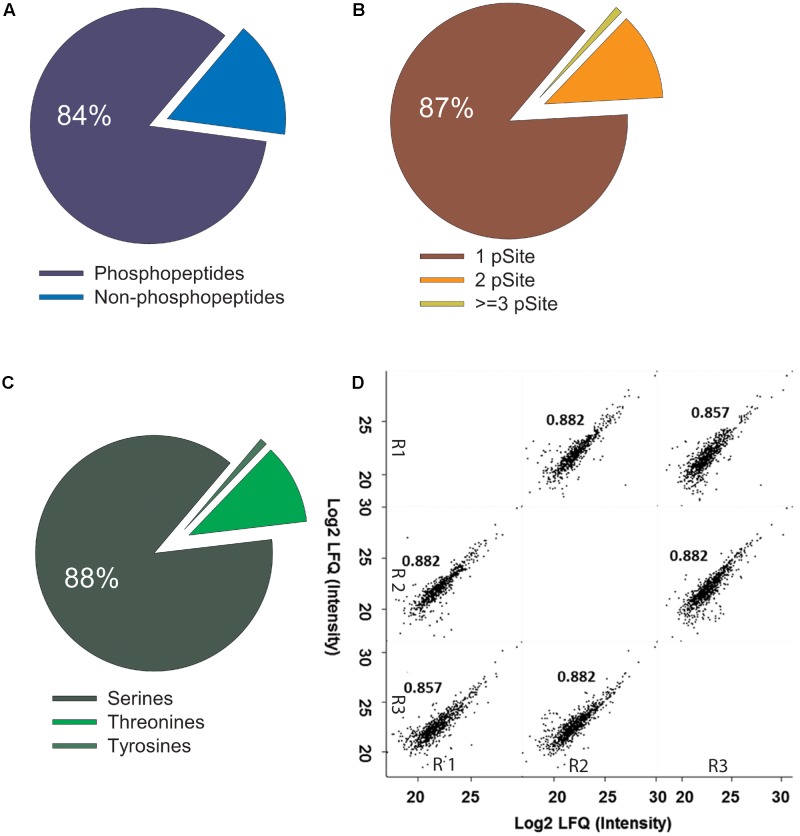
High specificity and reproducibility of phosphopeptide enrichment in ShortPhos phosphoproteomics workflow. **(A)** High percentage of phosphopeptides (84%) and low percentage of non-phosphopeptides identified in ShortPhos workflow. **(B)** Distribution of phosphorylated residues in the phosphopeptides: 1998 phosphopeptides with single phosphorylation site (87%), 278 with double phosphorylation sites (12%) and 10 (1%) with multiple phosphorylation sites. **(C)** Distribution of identified phosphorylation sites to serine (S), threonine (T) and tyrosine (Y). **(D)** Phosphopeptide intensity (log_2_ LFQ) comparison for LC-MS/MS replicates displayed a high label-free quantitative reproducibility between three biological replicates (R1, R2, and R3).

**FIGURE 3:**
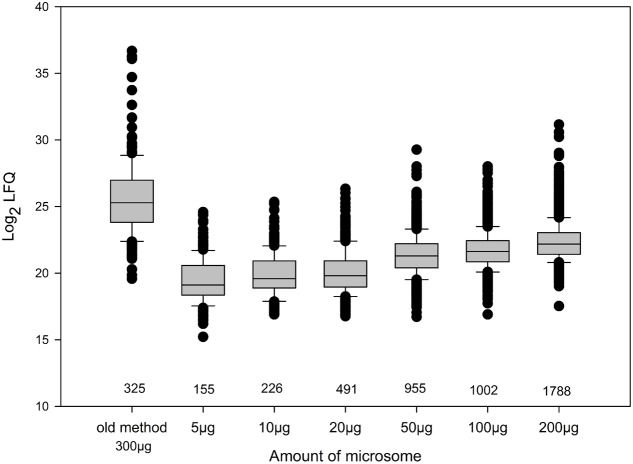
Distribution of phosphopeptide abundances (log_2_ LFQ) in previously published phosphoproteomics workflow and “ShortPhos” preparations with different amounts of starting material. LFQ = label free quantification.

To determine the phosphopeptide yield and sensitivity of the “ShortPhos” protocol, we digested different amounts of protein ranging from 5 to 200 μg MF proteins with trypsin, followed directly by the “ShortPhos” phosphopeptide enrichment procedure and single LC-MS/MS (**Supplementary Table S1**) runs. The resultant phosphoproteome comprised a total of 2833 unique peptides matching to 1263 proteins, among them 2260 phosphopeptides. As expected, high amounts of input MF proteins lead to higher peptide identifications (1796 phosphopeptides from 200 μg MF, while 165 phosphopeptides were identified from 5 μg MF; **Figure 3**). The previous workflow required four times more starting materials, and 1.5 times the amount of MF for the phosphopeptide enrichment, while identifying less than one-third of phosphopeptides (325 phosphopeptides, **Figure 3** and **Supplementary Table S3**). Generally, with all tested input amounts the “ShortPhos” workflow achieved a highly specific enrichment, a total of 84% of all identified unique peptides were phosphopeptides (2260, **Figure 2A** and **Supplementary Table S3**). The identified phosphopeptides were mostly singly (87%) or doubly phosphorylated (12%), and only rarely identified with three or more phosphorylation sites (<1%) (**Figure 2B**). Most of the identified phosphorylated amino acids were serines (88%), followed by threonines (11%) and tyrosines (1%) (**Figure 2C**). The quantitative reproducibility between three replicates was high with all combinations of sample-to-sample correlation yielded in *R*-values higher than 0.85 (**Figure 2D**).

We next compared the abundance distribution of log_2_-LFQ (label free quantitation) values (Cox et al., 2014) of the phosphopeptides resulting from the “ShortPhos” phosphopeptide enrichment protocol with the datasets obtained using the previous enrichment protocol (Wu and Schulze, 2015). In general, samples enriched following the “ShortPhos” protocol resulted in distributions of identified phosphopeptides to lower log_2_-LFQ values suggesting that more low-abundant phosphopeptides were identified in the new enrichment workflow (**Figure 3** and **Supplementary Table S4**). Phosphopeptide abundances in preparations from 5 to 20 μg MF displayed slightly lower log_2_-LFQ value distributions than enrichments from higher amounts of MF, reflecting the identified total peptide amount (**Figure 3**). We conclude that the “ShortPhos” workflow increased the yield of phosphopeptides per sample and particularly improved the ability for identification of lower abundance phosphopeptides even when using only very low amounts of starting membrane proteins.

### Functional Classification of Phosphopeptides from the “ShortPhos” Workflow

**FIGURE 4:**
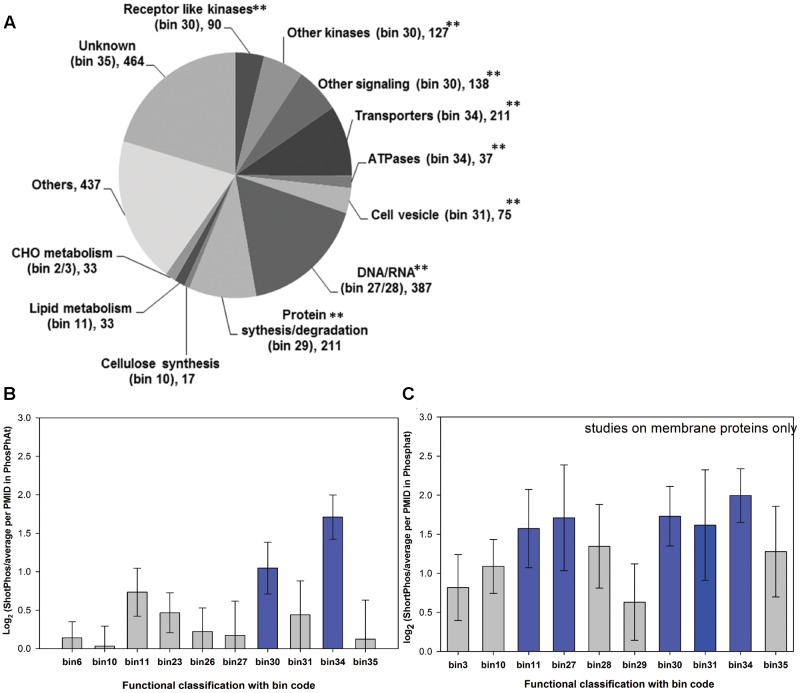
Functional classification of phosphopeptide data obtained from the “ShortPhos” workflow on liquid grown seedlings. **(A)** Identified phosphoproteins were classified into twelve groups based on MapMan bins. **(B)** Comparison (log_2_) between numbers of phosphoproteins in each bin identified by three biological replicates of “ShortPhos” compared to average numbers of protein in each bin identified in previous data sets published in the PhosPhAt database. **(C)** Comparison (log_2_) between numbers of phosphoprotein in each bin identified by “ShortPhos” compared to average numbers of protein in each bin from previous publication on membrane preparations in the PhosPhAt database.

The functional classification of the 2260 identified phosphopeptides obtained from the “ShortPhos” workflow was made based on MAPMAN (Thimm et al., 2004) using the “leading razor protein” (Cox et al., 2009) as a single protein identifier (**Figure 4A**). Phosphopeptides from signaling related proteins (bin 30) were highly enriched (*p* = 3.44 × 10–20, Fisher’s exact test, using Ath_AGI_Tair10 as a background), followed by transport-related proteins (bin 34) (*p* = 1.62 × 10–21, Fisher’s exact test) and cell vesicle transport (bin 31.4) related proteins (*p* = 1.12 × 10–15, Fisher’s exact test). Among the phosphopeptides from signaling proteins, 90 phosphopeptides were matched receptor kinases, including lower abundant kinases such as BAM1 (Barely any Meristem 1, AT5G65700) or BIR2 (BAK1-interacting receptor-like kinase 2, AT3G28450) and several membrane-associated kinases of the receptor-like cytoplasmic kinase family (e.g., BSKs). A total of 127 phosphopeptides were identified matching other kinases, such as calcium-dependent protein kinases (CPKs), SNRK1 (Sucrose non-fermenting (Snf)-2-related protein kinase), SNRK2, and MAP kinases (MAP2K, MAP3K and MAP4K). Further 142 phosphopeptides were identified from other signaling proteins, such as remorin family proteins and Rab proteins (ARA2 and ARA4). A total of 244 phosphopeptides were identified originating from PM located transporters, such as high abundant PM H^+^-ATPases (AHA1, AHA2, and AHA11), Ca^2+^ATPases (ACA8 and ACA1), and aquaporins (PIP3A, PIP3B, PIP2F, PIP2D, PIP2E and PIP2F). Phosphopeptides from lower abundant transporters were also identified, such as the potassium channels KUP1 (AT2G30070), KUP4 (AT4G23640), KUP5 (AT4G33530), and KAT3 (AT4G32650), or the chloride channel CLC-C (AT5G49890) and SLAH3 (AT5G24030). Phosphopeptides from proteins with function in cell vesicle trafficking included of clathrin family proteins and many syntaxin proteins (SYPs). Furthermore, a large set of phosphopeptides were identified from DNA/RNA binding proteins and proteins related to protein synthesis/degradation (e.g., ribosomes). Their identifications most likely can be attributed to the fact that proteins of these two functional groups are highly abundant in the cellular proteome and these proteins in addition are frequent found as co-purifying proteins in membrane preparations (Zauber et al., 2014).

We then compared the functional distribution of “ShortPhos” phosphopeptides to phosphoproteomics data sets in the PhosPhAt 4.0 database (Heazlewood et al., 2008; Durek et al., 2010) by calculating the ratio of phosphopeptide identifications in each Mapman bin category using “ShortPhos” versus the average identifications in that Mapman bin per publication hosted in PhosPhAt 4.0. The “ShortPhos” dataset of this study identified two-fold more signaling-related phosphopeptides (bin 30) and over three-times more transporter-related phosphopeptides (bin 34) compared to the average identifications per publication in PhosPhAt 4.0 (**Figure 4B**). Moreover, by comparing the functional distribution of identified phosphopeptides of “ShortPhos” with only the membrane protein studies in PhosPhAt 4.0 database, the increased coverage of phosphopeptides regarding signaling proteins (bin30), transporters (bin 34), lipid metabolism-related proteins (bin 11), vesicle trafficking and cytoskeleton (bin 31) as well as RNA-related proteins such as transcription factors (bin 27) was confirmed (**Figure 4C**).

### Subcellular Localization of Phosphopeptides from the “ShortPhos” Workflow

**FIGURE 5:**
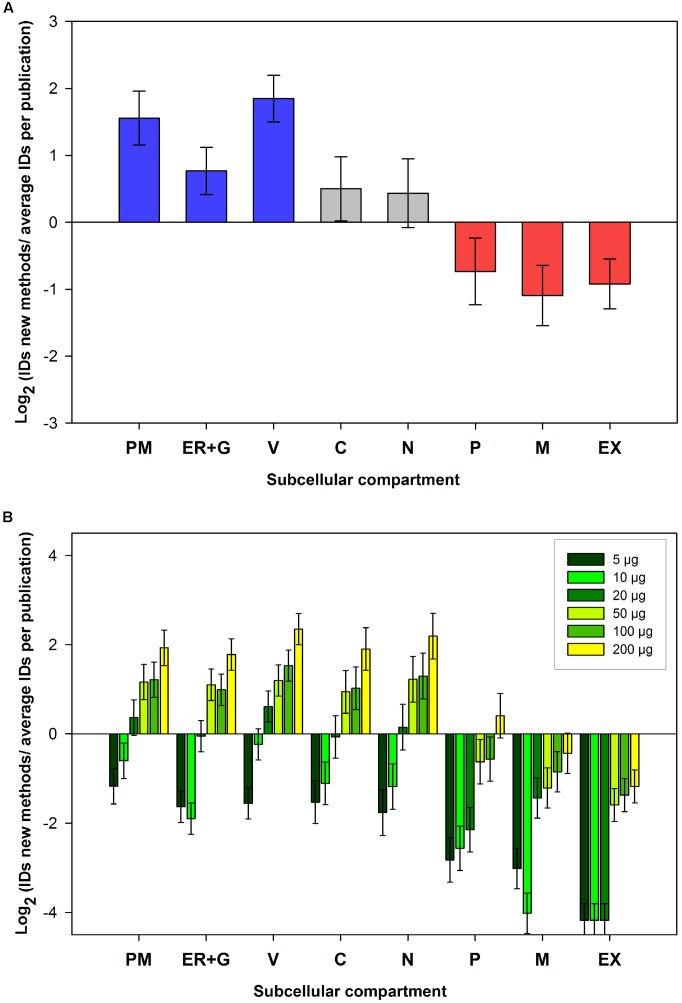
Subcellular localizations of phosphopeptide data obtained from the ShortPhos workflow. Corresponding proteins were assigned to their subcellular compartments based on SUBA3. **(A)** Comparison (log_2_) between numbers of phosphoprotein identified for each subcellular location by ShortPhos with the average number of phosphoproteins identified for each compartment in previous publications in the PhosPhAt database. **(B)** Comparison (log_2_) between phosphoproteins identified in different subcellular compartment from different amounts of starting materials compared with the average number of identified proteins from membrane protein data sets publications in PhosPhAt. PM: Plasma membrane, ER: Endoplasmic reticulum, G, Golgi apparatus; V, vacuole; C, cytosol; N, nucleus; P, plastid; M, mitochondrion; EX, extracellular.

Cellular membranes have important functions in metabolic compartmentation and signaling. Most proteins are localized to specific cellular compartments or subcellular membranes within the cell. In addition subcellular compartments and their membranes contain specific compositions of kinases (Van Wijk et al., 2014). Thus, an improvement of phosphoproteomics coverage of membrane proteins a prerequisite in our detailed understanding of cellular signaling processes. Therefore, phosphopeptides identified in the “ShortPhos” workflow and in the PhosPhAt 4.0 database were assigned to nine subcellular compartments (plasma membrane (PM), endoplasmic reticulum (ER), Golgi apparatus (G), vacuole (V), cytosol (C), nucleus (N), plastid (P), mitochondrion (M), and extracellular (EX)) based on the consensus compartment of their corresponding proteins in SUBA3 (Tanz et al., 2013). We then investigated the coverage of membrane proteins identified in the “ShortPhos” workflow compared to previously published data sets in PhosPhAt 4.0 database (**Figure 5A**). The “ShortPhos” method identified over three times more phosphopeptides from membrane proteins (PM, ER + G and V) compared to previously published data sets. Less than average numbers of phosphopeptides were identified by the “ShortPhos” methods from cytosolic, nuclear, plastidal, mitochondrial and extracellular compartments (**Figure 5A**).

Finally, we compared the number of identified phosphopeptides from each subcellular compartment using 5 μg up to 200 μg MF in the “ShortPhos” workflow and the number of identified average phosphopeptides obtained in previous publications on membrane phosphoproteomics (PM or MF) in PhosPhAt 4.0 database. The results revealed that with our optimized “ShortPhos” workflow, only 20 μg of MF as starting material is sufficient to identify more phosphopeptides from the PM and the vacuole than in previous membrane protein phosphoproteomics studies (**Figure 5B**). The use of higher amounts of MF for the “ShortPhos” workflow resulted in higher numbers of phosphopeptides, particularly from cellular membranes.

### Differential Phosphorylation Responses under Low and High Nitrate Supply

We explored to what extent the coverage of known kinase-substrate interactions was improved by using the “ShortPhos” workflow over previously published data sets using a nitrate starvation-resupply data set. Wild type roots from *Arabidopsis* hydroponic cultures (Schlesier et al., 2003) were stimulated with 0.2 mM or 5 mM nitrate for 15 min after 2 days of nitrogen starvation. In this data set, 3884 phosphopeptides were identified, out of these 833 phosphopeptides were from PM proteins, ER, Golgi or vacuolar proteins. A total of 320 phosphopeptides matching 264 proteins were found to be significantly up- or downregulated under at least one of the nitrate stimulation conditions (ANOVA *p*-value ≤ 0.05, **Supplementary Table S5**), and 311 phosphorylation sites of protein kinases were identified.

Under resupply of 0.2 mM nitrate for 15 min, 16 phosphorylation sites were significantly dephosphorylated, whereas 164 phosphorylation sites showed an increase in phosphorylation status (**Supplementary Figures S2A,C**). Under resupply of 5 mM nitrate, 69 phosphorylation sites decreased in phosphorylation status and 82 phosphorylation sites were found with increased phosphorylation (**Supplementary Figures S2B,C**). In most cases, both nitrate concentrations resulted in the same trend of response (up- or downregulation) but with stronger effects at high (5 mM) nitrate supply as concluded from a tendency to higher fold-changes (log_2_ values treatment/control) at 5 mM compared to 0.2 mM nitrate supply (**Supplementary Table S7**).

One protein among the few proteins with highly differential phosphorylation patterns under 0.2 mM nitrate supply and 5 mM nitrate supply was the CEP2 protein [peptide FADLT(ph)INEFK, T89], a cysteine endopeptidase 2. Supply of 5 mM nitrate resulted in significant higher phosphorylation of the CEP2 protein, while supply of 0.2 mM nitrate resulted in a dephosphorylated status of the protein. CEPs were shown to be involved in shoot to root signaling of the nitrogen status (Tabata et al., 2014). Thus, it is not surprising that under resupply of different nitrate concentrations, differential responses in CEP phosphorylation were observed. However, up to now the functional implications of CEP2 phosphorylation is not resolved.

Among the proteins with highly differential phosphorylation status at 0.2 mM and 5 mM nitrate supply were also several splicing factors, such as RSZ33, RSP31, or RSP35 which were differentially phosphorylated at several sites (**Supplementary Table S5**). It was shown previously that RNA stability is affected by the nitrate status of plants (Sakamoto et al., 2012), and thus we can expect proteins with functions in RNA processing under high nitrate supply to be differentially phosphorylated compared to low (0.2 mM) nitrate supply.

### Identified Kinases-Substrate Relationships

**FIGURE 6:**
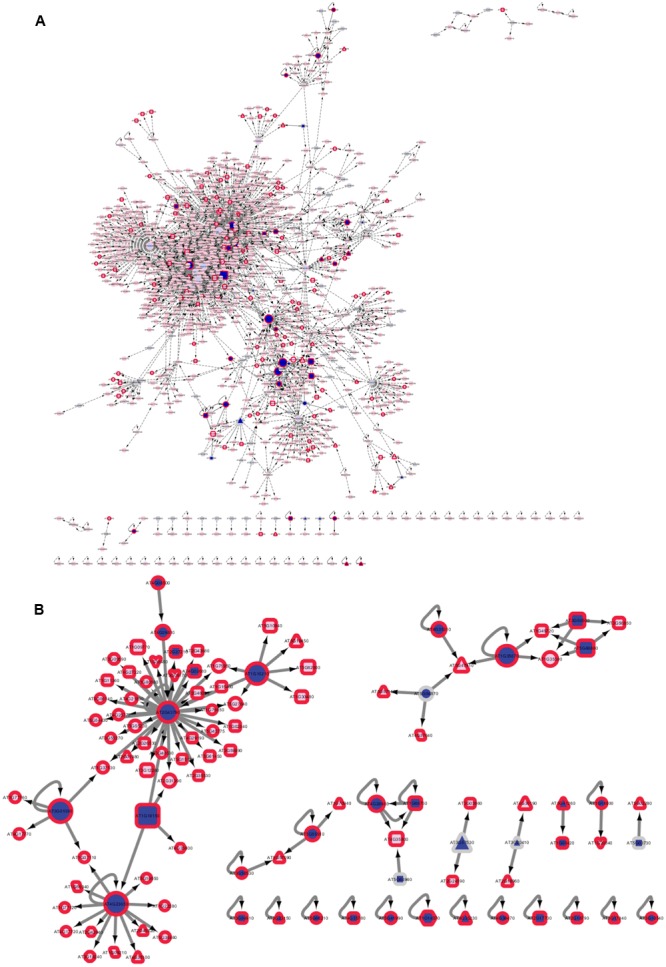
Kinase-Target relationships in a ShortPhos dataset. **(A)** Known kinase-substrate interactions downloaded from PhosPhAt with highlighted proteins identified with at least one phosphopeptide. **(B)** Network of identified kinases and their identified substrates in nitrate-induced signaling. Dark blue: identified kinase. Dark red border: identified substrate. Blue: Kinase, red: target. Darker color indicates identification.

A total of 2346 experimentally confirmed kinase-substrate relationships of *Arabidopsis* kinases were collected from published sources (Zulawski et al., 2013), but only 168 of these known kinase-substrate relationships involved protein kinases within or at the PM (**Figure 6A**). Despite the incompleteness, the existing known kinase-substrate network was used as benchmark to compare phosphopeptide enrichment workflows by mapping the identified proteins onto this kinase-substrate network. For 42 of these identified phosphorylated protein kinases at least one substrate is already known (Zulawski et al., 2013), and 161 known phosphorylated substrate proteins were identified. Mapping the identified kinases and substrates onto a known kinase-substrate network identified 113 known kinase-substrate interactions (**Supplementary Table S6**). Out of 23 kinase-substrate interactions involved kinases within or at the PM (**Figure 6B**). The number of known kinase-substrate interactions identified using the “ShortPhos” workflow exceeded by more than four times the number identified kinase-substrate relationships (only 25, **Supplementary Figure S1**) in previously published nitrate-starvation experiments (Engelsberger and Schulze, 2012). This is to be expected, as with higher overall phosphoproteome coverage by the “ShortPhos” workflow also the coverage of kinase-substrate pairs was increased.

### Nitrate-Induced Metabolic Responses

Specifically, the “ShortPhos” prepared nitrate-induced data set contained several highly relevant kinase-substrate pairs (**Supplementary Table S6**) and nitrate-induced phosphorylation sites (**Supplementary Table S7**). For example, nitrate reductase 1 (NIA1) and nitrate reductase 2 (NIA 2) were identified with their regulatory phosphorylation sites [peptides SVS(ph)SPFM(ox)NTASK and SVS(ph)TPFMNTTAK] which were significantly down-regulated under high (5 mM) nitrate supply, in agreement with previous results (Engelsberger and Schulze, 2012). NIA1 and NIA2 were dephosphorylated leading to activation of nitrate assimilation by nitrate reductase (Bachmann et al., 1996). A kinase known to phosphorylate NIA1 and NIA2, the SnRK1 kinase KIN10 was found to be phosphorylated at peptide MHPAESVAS(ph)PVSHR. Phosphorylation at this site was highly increased by both high and low nitrate supply, but the identified S387 was not the known activation site controlled by SnRK-activating kinase (Glab et al., 2017). Thus, a high nitrate supply did significantly alter kinase KIN10 phosphorylation, but with yet unknown effect on its activity. Instead, a phosphorylation site S(ph)TVGTPAYIAPEILLR at S177 of SnRK2.2 located in the kinase activation loop was increased in phosphorylation at 0.2 mM nitrate resupply. Another doubly phosphorylated peptide covering serines S623 and S265 was identified for NIA2 with significantly increased abundance under high (5 mM) nitrate supply. However, the functions of these phosphorylation sites are unknown.

An interaction of increased nitrate supply with carbon metabolism was identified by decreasing phosphorylation of sucrose phosphate synthase SPS1F at sites S734 and S738 under 5 mM nitrate supply. These sites are already known regulatory phosphorylation sites, and the de-phosphorylated SPS enzyme is known to be more active (Huber and Huber, 1996). Thus, nitrate resupply not only leads to an increased activity of nitrate assimilation by dephosphorylation of nitrate reductases NIA1 and NIA2, but also to an increased sucrose phosphate synthase activity by dephosphorylation of SPS.

### Nitrate-Induced Transport Processes

Nitrate uptake is coupled to protons (Parker and Newstead, 2014). Therefore, we were particularly interested in nitrate-induced regulatory phosphorylation sites of proton ATPases AHA1 and AHA2. So far, for each of the major plant proton ATPases, three regulatory phosphorylation sites were identified. Phosphorylation at S898 (AHA1) and S932 (AHA2) is known to inhibit proton pump activity, while T883/T881 and T948/T980 (AHA1/AHA2) are known phosphorylation sites of proton pump activation (Rudashevskaya et al., 2012). Known receptor kinases regulating the proton pump activity, such as FERONIA (Haruta et al., 2014) or PSY1R (Fuglsang et al., 2014) were not identified in the nitrate-induced data set. Instead, four other receptor kinase phosphopeptides were found (**Supplementary Table S7**), suggesting that under nitrate supply, a kinase other than FERONIA or PSY1R may regulate AHA1 and/or AHA2. A soluble kinase which is known to phosphorylate AHA1 and AHA2 is CPK9 (Lino et al., 1998), but the precise site of action is yet unknown. Previous work indicated that AHA1 and AHA2 are inhibited by CPK9 (Lino et al., 1998). We identified three phosphopeptides of CPK9, but only one identified phosphopeptide, AAAAAPGLS(ph)PK (Ser-69), was significantly up-regulated under both nitrate conditions. However, it is yet unknown, how these phosphorylation sites affect CPK9 activity.

Nitrate transporter NRT2.1 was identified with two phosphorylation sites at S28 and S11 (**Supplementary Table S7**). The phosphorylation of peptide EQSFAFSVQS(ph)PIVHTDK was previously shown with decreasing phosphorylation status upon nitrate resupply (Engelsberger and Schulze, 2012). Here, we confirm dephosphorylation at high (5 mM) nitrate within 15 min, and similar but lesser dephosphorylation also at low (0.2 mM) nitrate. In contrast, increased phosphorylation of (ac)GDSTGEPGSS(ph)MHGVTGR was observed at both nitrate concentrations. However, the roles of these phosphorylation sites still remains to be elucidated and also the kinase responsible for phosphorylation of the NRT2.1 is yet unknown. In our data set, 31 phosphopeptides matching 27 kinases of different families were identified as significantly up- or downregulated at either nitrate supply condition (**Supplementary Table S7**). These kinases, among them four receptor kinases, could be candidates for regulation of nitrate uptake via phosphorylation of NRT2.1.

### Nitrate-Induced Kinase Cascades

In a subnetwork of known kinase-substrate interactions, a MAP kinase cascade was identified consisting of MAP triple kinase MEKK1, MAP kinase MKK2, and MAP kinase MPK6 (**Figure 6A** and **Supplementary Table S7**). A doubly phosphorylated peptide S(ph)LEFPEPT(ph)SFR of MEKK1 was found significantly down-regulated under high (5 mM) nitrate. Two phosphopeptides were identified for MKK2, FLTQSGT(ph)FKDGDLR and IISQLEPEVLS(ph)PIKPADDQLSLSDLDMVK, both down-regulated under high nitrate. MPK6 was identified with the regulatory activating motif (pT)E(pY), covered by the peptide VTSESDFMT(ph)EY(ph)VVTR, which was also significantly down-regulated under high nitrate supply. Therefore, our data suggest that a MAP-kinase cascade involving MPK6 is inactivated (dephosphorylated) under high nitrate supply after 15 min. In contrast, MPK1 [GQFMT(ph)EY(ph)VVTR], was identified with a significant increase in phosphorylation at the activating TEY motif at high nitrate supply suggesting MPK1 to be activated under low affinity nitrate uptake conditions (e.g., 5 mM nitrate). MPK8 [VSFNDAPTAIFWTDY(ph)VATR] was also found with a slightly increased phosphorylation at both nitrate resupply conditions. An involvement of Mitogen-activated protein kinase-signaling cascades in nutrient signaling was recently reviewed (Chardin et al., 2017), confirming the identifications in our data set.

## Conclusion

Plant membranes, particular the PM, have a key function in sensing and transducing signals from the environment, such as nutrients or environmental status (Osakabe et al., 2013). The analysis of the plant membrane phosphoproteome has already revealed novel kinase-target relationships in context of nutrient signaling (Engelsberger and Schulze, 2012; Wu et al., 2013) or hormone signaling (Caesar et al., 2011; Fuglsang et al., 2014; Haruta et al., 2014). Thus, to more efficiently elucidate these regulatory patterns within membranes, highly efficient methods for phosphopeptide enrichment from low amounts of protein are required. Here we introduced a cost-effective high-throughput method, called “ShortPhos,” for phosphoproteome studies on plant membrane proteins. Our “ShortPhos” workflow is based on three major improvements: Firstly, we applied an optimized MF isolation method instead of traditional PM isolation, which saved much hands-on time and cost. The MF isolation buffer described here does not contain any detergent or SDS, instead, a Dounce Homogenizer-based MF homogenization was applied. This enables a directly further use of the MF for phosphopeptide enrichment without the need to remove interfering detergents or SDS. Secondly, we omitted peptide fractionation after trypsin digestion, which saved running time on the mass spectrometer, and instead performed single MS runs with extended chromatographic gradients. Thirdly, the “ShortPhos” phosphopeptide protocol has greatly improved the efficiency of phosphopeptide enrichment, yielding 84% of phosphopeptides in each LC-MS/MS run. Overall, the “ShortPhos” workflow improved the ability to identify low abundant phosphopeptides (**Figure 3**) and the coverage of membrane proteins, in particular transport-related proteins and signaling related proteins was highly improved compared to previously published data sets (**Figures 4**, **5**). Thus, the “ShortPhos” workflow leads to a significantly improved coverage of known kinase-substrate relationships (**Figure 6**). Taken together, “ShortPhos” workflow can produce high quality phosphoproteomics data for reproducible quantification, even if researchers have limited amount of starting material. Furthermore, since the “ShortPhos” workflow consists of a membrane enrichment step and a phosphopeptide enrichment step, each of these optimized protocols can be used individually or in combination with other protocols, simply by adapting with either different organelle preparations (other than membranes) or with other peptide enrichment protocols (other than phosphopeptides).

## Author Contributions

XW and WS provided experimental design. XW carried out the main lab work (seedling liquid culture, optimization of phosphoproteomics experiments, LC-MS/MS analysis), data analysis, and mainly wrote the manuscript. WS provided funding support, helped with MS analysis of samples, phosphoproteomics data analysis (kinase-substrates network), and manuscript revision. LX performed the data analysis manuscript revision and part of figure preparation. HP-O optimized the membrane protein isolation protocol. L-CC and ZL prepared the materials for the nitrate starvation and resupply experiment. All the authors approved the final manuscript.

## Conflict of Interest Statement

The authors declare that the research was conducted in the absence of any commercial or financial relationships that could be construed as a potential conflict of interest.

## Supplementary Material

FiGURE S1 Kinase-Target relationships identified in previous data sets (Engelsberger and Schulze, 2012). Dark Blue: identified kinase. Dark Red border: identified substrate. Blue: Kinase, red: target. Darker color indicates identification.

FiGURE S2 Phosphorylation responses under low and high nitrate supply. **(A)** Volcano plot compare phosphopeptides responses at 0.2 mM nitrate supply and nitrate starvation. **(B)** Volcano plot compare phosphopeptides responses at 5 mM nitrate supply and nitrate starvation. **(C)** Overlap of significantly up- or down-regulated phosphorylation sites at 0.2 mM or 5 mM nitrate supply.

TABLE S1 LC-MS/MS settings are standard setting for EASY-nLC 1,000 liquid chromatography system coupled to Q ExactivePlus mass spectrometer via EASY-Spray ion source using EASY-Spray analytical column (25 cm × 75 μm inner diameter).

TABLE S2 MaxQuant (Cox et al., 2009) setting for phosphopeptides identification and ion intensity quantification from raw mass spectrometry data.

TABLE S3 Comparison of the numbers of phosphopeptides identified in this study and previous studies using MF preparations.

TABLE S4 LFQ values for different phosphopeptides obtained from different amounts of starting material.

TABLE S5 Full list of identified phosphopeptides in the nitrate induction experiments including LFQ value ratios (log2 NO3 resupply/NO3 starvation) for 0.2 mM and 5 mM nitrate supply.

TABLE S6 List of identified kinase-substrate pairs.

TABLE S7 List of phosphopeptides of selected substrate proteins and all kinases with significant nitrate-induced phosphorylation changes. Phosphorylation changes are displayed as log_2_ ratios of LFQ values obtained for nitrate induced conditions (0.2 or 5 mM nitrate resupply for 15 min) versus nitrate starved controls. Averages of three biological replicates are shown.

TABLE S8 Full information of identified peptide in this study.
