# Supplementary figures and images for: Highly Efficient Single-Step Enrichment of Low Abundance Phosphopeptides from Plant Membrane Preparations
Source: Front Plant Sci. 2017 Sep 27;8:1673. doi: 10.3389/fpls.2017.01673 (PMC5632542; doi:10.3389/fpls.2017.01673)

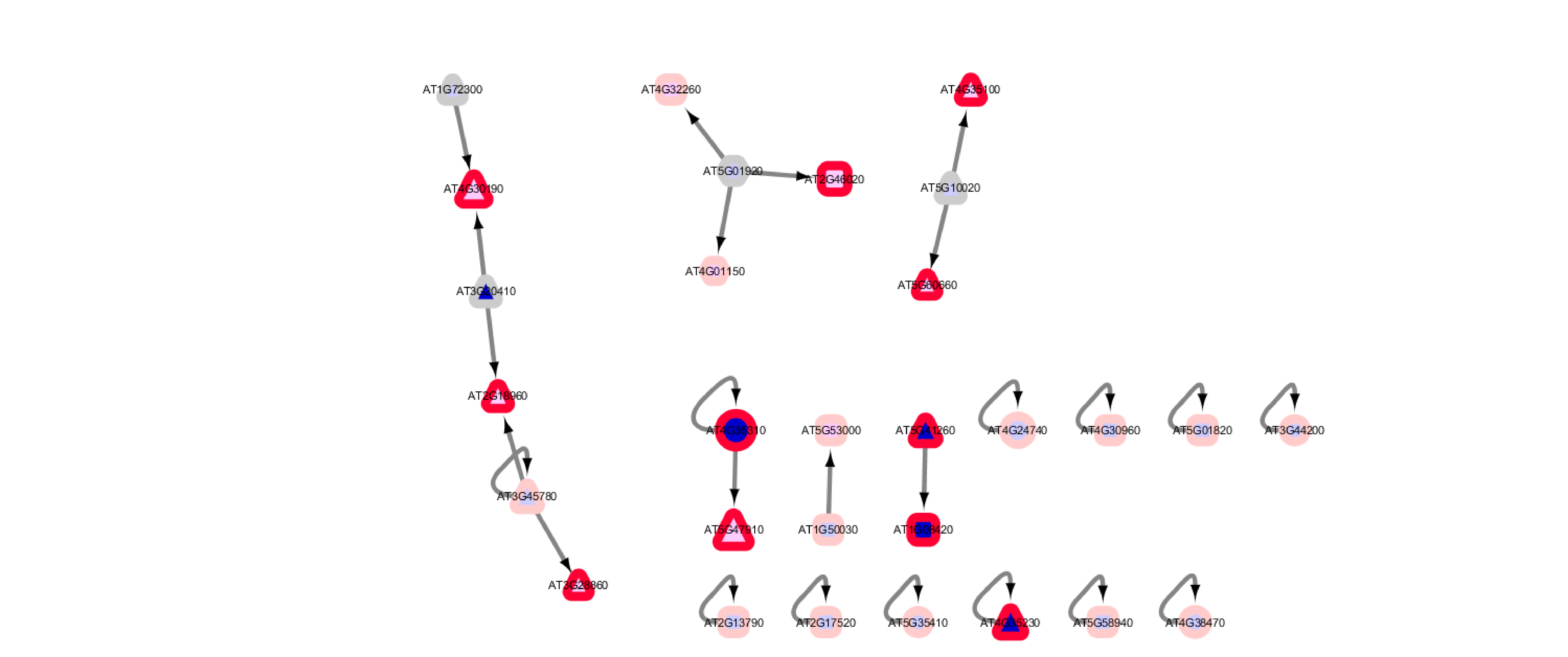

Supplement: FiGURE S1 — Kinase-Target relationships identified in previous data sets (Engelsberger and Schulze, 2012). Dark Blue: identified kinase. Dark Red border: identified substrate. Blue: Kinase, red: target. Darker color indicates identification. [file Image_1.JPEG]

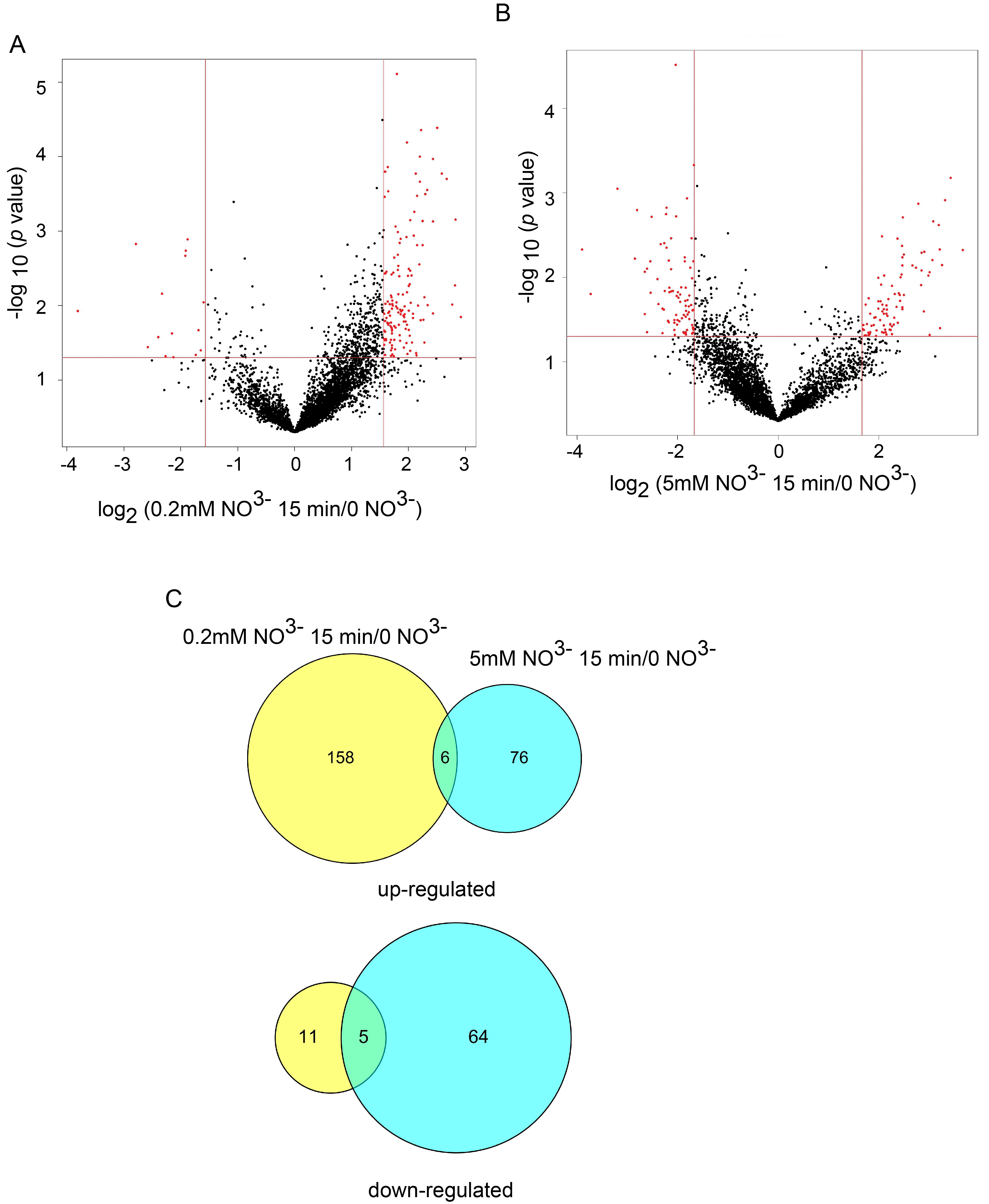

Supplement: FiGURE S2 — Phosphorylation responses under low and high nitrate supply. (A) Volcano plot compare phosphopeptides responses at 0.2 mM nitrate supply and nitrate starvation. (B) Volcano plot compare phosphopeptides responses at 5 mM nitrate supply and nitrate starvation. (C) Overlap of significantly up- or down-regulated phosphorylation sites at 0.2 mM or 5 mM nitrate supply. [file Image_2.JPEG]
