# Supplementary material for: Highly Efficient Single-Step Enrichment of Low Abundance Phosphopeptides from Plant Membrane Preparations
Source: Front Plant Sci. 2017 Sep 27;8:1673. doi: 10.3389/fpls.2017.01673 (PMC5632542; doi:10.3389/fpls.2017.01673)
Supplement: TABLE S1 — LC-MS/MS settings are standard setting for EASY-nLC 1,000 liquid chromatography system coupled to Q ExactivePlus mass spectrometer via EASY-Spray ion source using EASY-Spray analytical column (25 cm × 75 μm inner diameter). [file Tables_1-3.PDF]

Supplementary Material

Highly efficient single-step enrichment of low abundance phosphopeptides from plant membrane preparations

Xu Na Wu[1*], Lin Xi[1], Heidi Pertl-Obermeyer[1,2], Zhi Li[1], Liang Cui Chu[1], Waltraud X. Schulze[1]

[1] Department of Plant Systems Biology, University of Hohenheim, 70599 Stuttgart, Germany

[2] present address: Molecular Plant Biophysics and Biochemistry, Department of Molecular Biology, University of Salzburg, Billrothstrasse 11, 5020 Salzburg, Austria

*Correspondence:
Dr. Xu Na Wu
xwu@uni-hohenheim.de

Department of Plant Systems Biology

University of Hohenheim

70599 Stuttgart

Germany

Supplementary Table S1: LC-MS/MS settings are standard setting for EASY-nLC 1,000 liquid chromatography system coupled to Q ExactivePlus mass spectrometer via EASY-Spray ion source using EASY-Spray analytical column (25 cm × 75 μm inner diameter).

Instrument	Parameter	Setting
LC system	Sample loading	At maximum pressure 800 bar
	Gradient length(min)	135
	Gradient flow rate(nl/min)	250
	Linear gradient(percentage of LC solvent B in LC solvent A	5-35% for 115min<br>35%-45% for 15min<br>45-90% for 5min
	Column heater temperature(ºC)	35
Ion source	Spray voltage(kV)	2.1
	Capillary temperature(ºC)	250
Mass spectrometer:Full MS	Resolution (at m/z 400)	70,000
	Automatic gain control target(ions)	1,000,000
	Maximum injection time(ms)	120
	Scan range(m/z)	300-1,600
Mass spectrometer:dd-MS2	Resolution (at m/z 400)	35,000
	Automatic gain control target(ions)	500,000
	Maximum injection time(ms)	120
	Loop count	12
	Isolation window(m/z)	1.2
	TopN	12
	Scan range(m/z)	200-2,000
	Normalized collision energy	25
Mass spectrometer:general	Polarity	Positive
	Intensity threshold	100,000
	charge exclusion	unassigned, 1,8,>8
	Peptide match	preferred
	Dynamic exclusion(sec)	20

Supplementary Table S2: MaxQuant setting for phosphopeptides identification and ion intensity quantification from raw mass spectrometry data

Parameter type	Parameter	Settings
Group-specific parameters	Version	1.5.3.8(or newer)
	Type	Standard
	Multiplicity	1
	Enzyme	Trypsin/P
	Maximum missed cleavage	2
	Variable modification	Oxidation(M)<br>Acetylation(protein N-term)<br>Phosphorylation(STY)
	Label-free quantification	Fast LFQ
	First search p.p.m.	20
	Main search p.p.m.	4.5
	Match type	Match from and to
Globle parameter	Include contaminants	TRUE
	Fixed modification	Carbamidomethyl (C)
	Minimum peptide length(residue)	7
	Maximum peptide mass(Da)	4600
	PSM FDR	0.01
	Protein FDR	0.01
	Site decoy fraction	0.01
	Match between runs	TRUE
	Match time window(min)	1
	Alignment time window(min)	20
	MS/MS tolerance(Fourier trsnsform MS,p.p.m.)	20
	Top MS/MS peaks per 100Da	12
	Special amino acids	K R

Supplementary Table S3: Phosphopeptides identified in this study and previous studies using MF

REF.	Organism	Starting material	Starting MF	number of phosphopeptides
this study	Arabidopsis thaliana	1g	200µg	2177
Engelsberger, et al. 2012 TPJ, PMID 22060019	Arabidopsis thaliana	4g	200µg	652
Wu, et al. 2013 MCP, PMID 23820729	Arabidopsis thaliana	4g	300µg	479
Wu, et al. 2014 JPR, PMID 24924143	Arabidopsis thaliana	4g	300µg	709
